# Thyroid Storm Triggered by Rotavirus Infection in a 10-Year-Old Girl

**DOI:** 10.1155/crie/9960607

**Published:** 2025-01-31

**Authors:** Masazumi Miyahara, Shizuka Otsuki

**Affiliations:** ^1^Department of Pediatrics, Okanami General Hospital, 2711-1 Kaminoshou, Iga 518-0121, Mie, Japan; ^2^Department of Pediatrics, Kuwana City Medical Center, 3-11 Kotobukichou, Kuwana 511-0061, Mie, Japan

## Abstract

We encountered a case of Graves' disease in a pediatric patient who presented with thyroid storm (TS), the onset of which was triggered by rotavirus infection. Rotavirus is commonly associated with severe watery diarrhea, vomiting, fever, and dehydration—particularly in infants and young children. In more severe cases, it can also lead to altered consciousness and seizures. These symptoms can resemble those of TS, even in the absence of pre-existing hyperthyroidism. In cases of hyperthyroidism, these symptoms confirm the criteria for TS. Therefore, it is possible that some cases of TS are triggered and caused by gastrointestinal conditions such as rotavirus infection. Our case highlights the need for further investigation into the potential triggering pathogens in patients with TS to better clarify details regarding the patient's status or disease pathogenesis.

## 1. Introduction

Thyroid storm (TS) is a rare but life-threatening endocrine complication of thyrotoxicosis that is characterized by a hypermetabolic state with multiorgan dysfunction. It is associated with a mortality rate of 8%–30% [1]. Infections represent a well-known trigger of TS. Various infectious organisms trigger TS, primarily those associated with the respiratory tract [2]. By contrast, rotavirus infection causes severe gastrointestinal symptoms—including diarrhea, vomiting, and dehydration—that can be life-threatening in young children. It is considered a primary cause of acute gastroenteritis in children worldwide. According to the US Centers for Disease Control and Prevention, the most common symptoms of rotavirus infection include watery diarrhea, vomiting, fever, and stomach pain—all of which can lead to dehydration. The symptoms of dehydration (e.g., decreased urination, dry mouth and throat, dizziness, and unusual sleepiness or fussiness [3]) can mimic those of TS, even in patients without pre-existing hyperthyroidism. In patients with hyperthyroidism, these symptoms can meet the diagnostic criteria for TS. Thus, certain cases of TS may be precipitated by gastrointestinal conditions such as rotavirus infection. Herein, we report a case of Graves' disease in a pediatric patient who presented with TS that was ultimately found to have been triggered by a rotavirus infection.

## 2. Case Presentation

A previously healthy 10-year-old Japanese girl was admitted to our hospital because of excessive vomiting. Two days prior, she had developed generalized fatigue, frequent vomiting, and fever. She had also been experiencing fatigue following mild exercise, such as running or school-based physical activities, for ~4 months prior to her hospitalization. However, neither she nor her parents considered these symptoms unusual and simply waited to see whether they would resolve spontaneously. The patient had no significant past medical history prior to this event, and her family history was unremarkable. Upon admission, she exhibited pronounced fatigue and lethargy. Her consciousness level was E3 V4 M5 on the Glasgow Coma Scale, and she had temperature, heart rate, respiratory rate, and blood pressure readings of 37.3°C, 182 beats/min, 54 breaths/min, and 170/90 mmHg, respectively. Physical examination revealed mild exophthalmos characterized by subtle bilateral protrusion of the eyes with slight upper eyelid retraction and visible sclera above the iris, no conjunctival injection or chemosis, intact extraocular movements, and diffuse bilateral goiter. No palpable nodules, tenderness, or bruits were observed. Laboratory analysis results were as follows: white blood cell count, 5100 cells/µL (75% neutrophils); C-reactive protein, 1.19 mg/dL; aspartate aminotransferase, 61 IU/L; alanine aminotransferase, 54 IU/L; creatine phosphokinase, 98 mg/dL; blood urea nitrogen, 27.2 mg/dL; creatinine, 0.19 mg/dL; sodium, 136 mEq/L; potassium, 3.8 mEq/L; chloride, 98 mEq/L; total cholesterol, 86 mg/dL; and glucose, 85 mg/dL. Serum thyroid function tests revealed a suppressed thyroid-stimulating hormone (TSH) level of <0.00 mIU/L (reference range, 0.35–4.94 mIU/L), elevated free triiodothyronine (T3) levels of 16.22 pg/mL (reference range, 1.68–3.67 pg/mL), and free thyroxine (T4) levels of >6.00 ng/dL (reference range, 0.70–1.48 ng/dL). Her TSH receptor antibody (TRAb) level was significantly elevated at 62.9 IU/L (reference range, <1.5 IU/L). A rapid fecal antigen test for rotavirus was positive. Thyroid echography revealed diffuse swelling and increased internal blood flow in the thyroid gland. Its right lobe measured 5.5 cm in length, 1.8 cm in width, and 1.2 cm in thickness, while the left lobe measured 5.0 cm in length, 2.3 cm in width, and 1.3 cm in thickness. An echocardiogram revealed no evidence of cardiomyopathy or reduced ejection fraction (EF), with an EF ratio of 64.5% (normal values EF for children, 56%–78%). However, mild mitral valve regurgitation was noted. The patient's score of 60 points (5 points for a fever of 37.2–37.7°C, 25 points for tachycardia of ≥140 beats/min, 20 points for moderate central nervous system disturbance, and 10 points for positive precipitating event) met the Burch-Wartofsky criteria for TS [4]—wherein a score of ≥45 points is highly indicative of TS. We, therefore, diagnosed the patient with TS triggered by rotavirus infection. Intravenous fluid therapy with hydrocortisone (100 mg once per day), potassium iodide solution (30 mg/day for 4 consecutive days), and propylthiouracil (25 mg/kg/day [total, 750 mg/day] for 4 consecutive days) was administered. The day after her admission, the patient's generalized fatigue had improved significantly, and her vomiting ceased. Her heart rate slowed to ~120 beats/min and finally stabilized at ~100 beats/min by her third day of hospitalization. The patient experienced muddy diarrhea 2–3 times per day for 2 days following her admission, which subsequently resolved. Her propylthiouracil was switched to thiamazole (1 mg/kg/day [total, 30 mg/kg/day]) on the 5th day of her hospitalization. A thyroid function test on the 8th day revealed: TSH, 0.00 mIU/L; free T3, 9.99 pg/mL; and free T4, 2.57 ng/dL. As her symptoms remained stable without deterioration, the patient was discharged on the 9th day and transitioned to outpatient follow-up care while continuing to take thiamazole. Her thyroid function normalized ~3 months later.

## 3. Discussion

TS is a life-threatening exacerbation of thyrotoxicosis, and its delayed diagnosis can lead to a high chance of mortality. Our patient presented with hyperthyroidism accompanied by elevated TRAb levels, involving failures of multiple organs and fulfilling the diagnostic criteria for Grave's disease with TS. TS can be triggered by a number of factors, including infections. According to a nationwide survey conducted in Japan, the most common illness triggering TS was infection, particularly of the upper respiratory tract [2]. However, specific infectious agents have not yet been elucidated for this type of TS. There is currently limited documentation in the literature regarding which infectious agents can act as triggers, leading to a lack of comprehensive understanding of TS. Previously reported causative organisms include only respiratory pathogens such as respiratory syncytial virus (RSV), influenza virus, *Streptococcus pyogenes* (*S. pyogenes*), and SARS-CoV-2 [5]. Since this case occurred prior to the emergence of SARS-CoV-2, the patient was not tested for it. Testing for RSV, influenza, or *S. pyogenes* was also not performed, as the patient exhibited no respiratory symptoms and had not been present during any known outbreaks of these pathogens. Given the patient's gastrointestinal symptoms, a positive test result for rotavirus antigen in the stool, and a concurrent diagnosis of thyrotoxicosis, we concluded that our patient developed TS triggered by gastroenteritis that was attributable to rotavirus infection. Exophthalmos was observed upon the patient's admission to hospital, suggesting a pre-existing state of hyperthyroidism that subsequently progressed to TS triggered by an infection. The mechanisms through which infections induce abnormal thyroid function remain unclear; however, infection-triggered cytokine production can lead to disturbances in thyroid function [6]. Antigenemia and hypercytokinemia are commonly present in children with rotavirus infection. Although cytokine levels were not measured in our patient, excess cytokines produced in response to the rotavirus infection may have affected her thyroid function. Gastrointestinal symptoms are relatively common in TS; therefore, it is important not to overlook the possibility of an underlying gastrointestinal infection acting as a trigger. Fortunately, our patient developed typical symptoms and findings indicating TS shortly after the onset of her gastrointestinal symptoms, facilitating a timely diagnosis of TS. Her triggering condition of rotavirus infection is also a self-limiting disease that does not typically require specific treatment. However, if there had been a longer interval between the onset of the patient's infection and her development of TS, or if the gastrointestinal symptoms caused by her infection had been more severe, it is uncertain whether TS would have been appropriately considered and managed. It is important to recognize that common infections can serve as triggers for TS and to remain vigilant for this possibility in clinical practice. Clinicians should consistently strive to identify the causative pathogens that trigger TS, as this may lead to a more thorough understanding of its pathophysiology and pathogenesis.

The main limitation of this study is that the possibility of rotavirus-associated encephalitis or encephalopathy has not been completely ruled out. According to the Japan Thyroid Association's guidelines for TS management, cases are excluded in the presence of other underlying diseases determined to be clearly responsible for fever, impaired consciousness, heart failure, or liver disorders [7]. Therefore, the exclusion of rotavirus-associated encephalitis or encephalopathy, which can both present with similar symptoms to TS, must be performed using cerebrospinal fluid tests, electroencephalography, and brain magnetic resonance imaging. As our patient's level of consciousness rapidly improved following treatment for TS, further investigations were deemed unnecessary at the time. However, given that the steroids used to treat TS can also alleviate encephalitis and encephalopathy, such investigations would be required for a definitive differentiation of other potential causes of encephalopathy to ensure patient safety.

We encountered a case of Graves' disease in a pediatric patient who presented with TS that was triggered by a rotavirus infection. The gastrointestinal symptoms and altered consciousness observed in TS may also be present in cases of rotavirus gastroenteritis, making differentiating between the two conditions potentially challenging. In some cases, TS may be triggered by gastroenteritis—such as that caused by rotavirus. When TS develops, it is crucial to investigate and accurately differentiate any underlying illnesses.

## Data Availability

The datasets used and/or analyzed during the current study are available from the corresponding author upon reasonable request.
